# On mobility trends analysis of COVID–19 dissemination in Mexico City

**DOI:** 10.1371/journal.pone.0263367

**Published:** 2022-02-10

**Authors:** Kernel Prieto, M. Victoria Chávez–Hernández, Jhoana P. Romero–Leiton

**Affiliations:** 1 Instituto de Matemáticas, Universidad Nacional Autónoma de México, Mexico, México; 2 Facultad de Ingeniería Mecánica y Eléctrica, Universidad Autónoma de Nuevo León, San Nicolás de los Garza, Mexico, México; 3 Facultad de Ingeniería, Universidad Cesmag, Pasto, Colombia; Columbia University, UNITED STATES

## Abstract

This work presents a tool for forecasting the spread of the new coronavirus in Mexico City, which is based on a mathematical model with a metapopulation structure that uses Bayesian statistics and is inspired by a data-driven approach. The daily mobility of people in Mexico City is mathematically represented by an origin-destination matrix using the open mobility data from Google and the Transportation Mexican Survey. This matrix is incorporated in a compartmental model. We calibrate the model against borough-level incidence data collected between 27 February 2020 and 27 October 2020, while using Bayesian inference to estimate critical epidemiological characteristics associated with the coronavirus spread. Given that working with metapopulation models leads to rather high computational time consumption, and parameter estimation of these models may lead to high memory RAM consumption, we do a clustering analysis that is based on mobility trends to work on these clusters of borough separately instead of taken all of the boroughs together at once. This clustering analysis can be implemented in smaller or larger scales in different parts of the world. In addition, this clustering analysis is divided into the phases that the government of Mexico City has set up to restrict individual movement in the city. We also calculate the reproductive number in Mexico City using the next generation operator method and the inferred model parameters obtaining that this threshold is in the interval (1.2713, 1.3054). Our analysis of mobility trends can be helpful when making public health decisions.

## Introduction

The coronavirus disease 2019 (COVID-19) is caused by a novel coronavirus. The coronaviruses are a family of viruses that cause infection in humans and animals. The diseases that are by a coronavirus are zoonotic [1]. In particular, the coronaviruses that affect humans (HCoV) can produce clinical symptoms, such as the Severe Acute Respiratory Syndrome (SARS) viruses and Middle East Respiratory Syndrome (MERS-CoV) [2]. COVID-19 was first identified amid an outbreak of respiratory illness cases in Wuhan City, Hubei Province, China. This disease was initially reported to the WHO on 31 December 2019. On 11 March 2020, the WHO declared COVID-19 to be a global pandemic [3]. From the beginning of the epidemic to 21 January 2021, more than 97,890,676 cases and 2,094,459 deaths have been reported globally.

The first case of COVID-19 in South America was registered in Brazil on 26 February 2020. The first death from this infection in this region was announced in Argentina on 7 March 2020. The virus then arrived in Mexico, where by 21 January 2021 there have been almost 1,688,944 confirmed cases and 144,371 deaths.

To date, many researchers around the world have focused on understanding the transmission dynamics of COVID-19 disease using mathematical and statistical models and methods, see for example [4–12]. In this work, we will focus on those models that incorporate information on human movement. The relationship between human mobility and the transmission of coronavirus disease in the United States has been studied in [13, 14]. Metapopulation models are not only among the simplest spatial models but they are also the most applicable to modelling many human diseases [15]. The metapopulation concept is to subdivide the entire population into distinct *sub-populations*, each of which has independent dynamics together with limited interaction between the sub-populations. This approach has been used to great effect within the ecological literature [16] and it has recently been used to model the spread of COVID-19; see, for example, [17–21].

In this work, we calibrate the metapopulation model proposed by Li et al. in [21], similar to [22], using incidence data reported in [23]. Consequently, we first describe the mathematical model that we have used; then, we compute the number of trips that are produced and attracted in each borough of Mexico City using data about these trips in 2017 [24], which then we combine with the rates of reduction or increase in mobility during the pandemic reported by Google [25] and the government of Mexico City [26]. Later, by using Bayesian inference, we solve the associated inverse problem to predict the dynamics of the spread of cases, similar to the following references [27–33]. Our conclusions are presented in the last section.

## Computation of the mobility matrices

**Fig 1:**
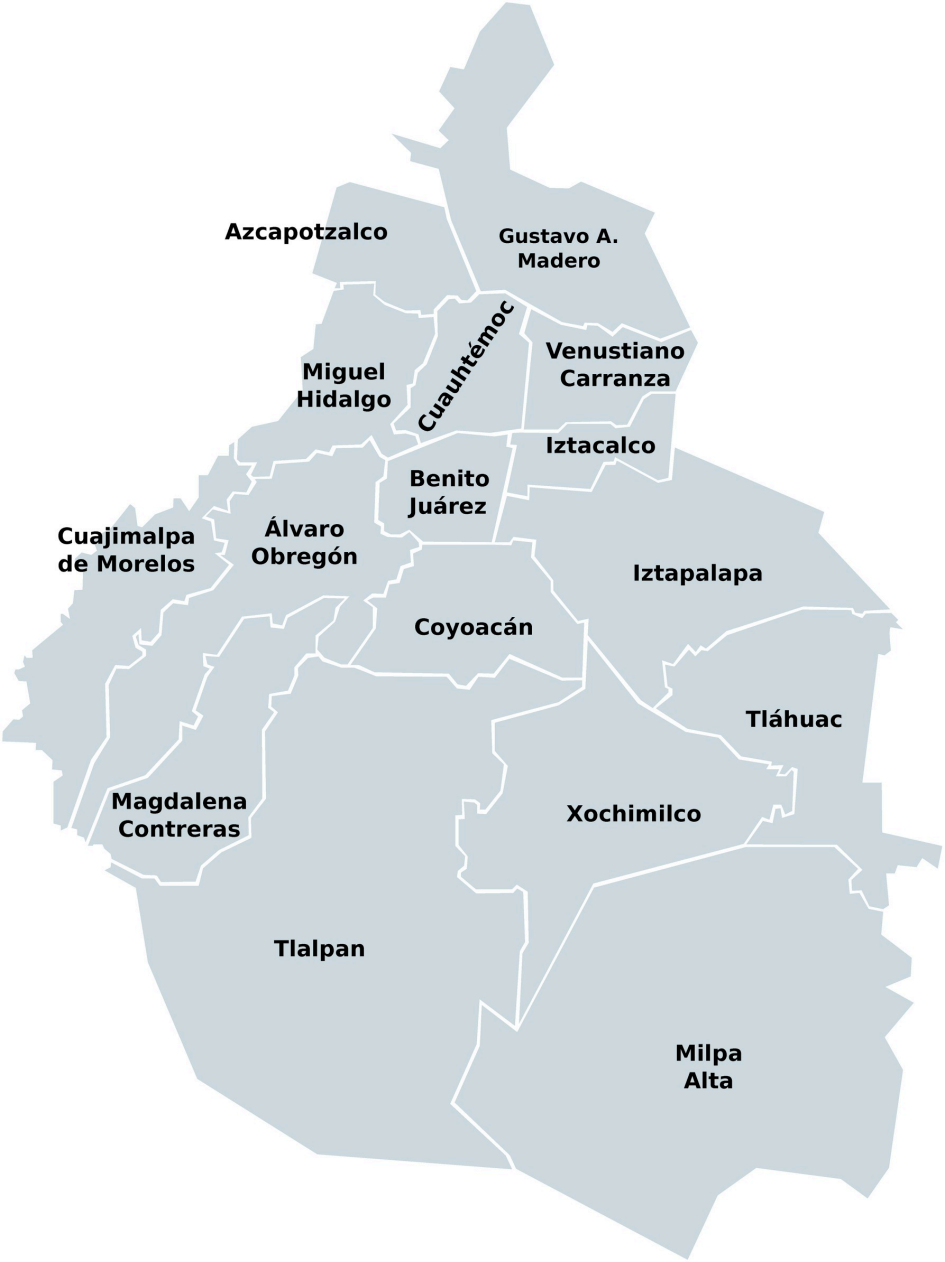
Boroughs of Mexico City.

To incorporate mobility in the transmission model, the produced and attracted trips in the boroughs of Mexico City are considered (see Fig 1 and Table 1). Mexico City is the capital of Mexico. It has around 9 million inhabitants and a floating population of over 22 million, who are composed of daily commuters and international visitors. Mexico City is among the top 10 most crowded cities in the world [34]. It also has a large number of corporate headquarters and a large transport network, which is composed of 20 different modes of transport.

**Table 1:** Boroughs of Mexico City.

Id.	Name	Id.	Name	Id.	Name	Id.	Name
1	Azcapotzalco	5	Iztacalco	9	Álvaro Obregón	14	Cuauhtémoc
2	Coyoacán	6	Iztapalapa	10	Tláhuac	15	Miguel Hidalgo
3	Cuajimalpa de	7	La Magdalena	11	Tlalpan	16	Venustiano
	Morelos		Contreras	12	Xochimilco		Carranza
4	Gustavo A. Madero	8	Milpa Alta	13	Benito Juárez		

Mobility between the zones in Table 1 is represented in a two-dimensional arrangement, which is known as the origin-destination matrix (O-D matrix) **M** = {*M*_*ij*_}, *i*, *j* = 1, …, 16, where *M*_*ij*_ represents the number of trips from zone *i* to zone *j*. Origin-destination matrices are usually obtained every 10 years from surveys. In Mexico City, the last survey was carried out in 2017 [24]. The information available identifies, among other things: if the trip was made on a weekday or if it was made during the weekend, the transport mode used, the purpose and the time. It is important to notice that, due to the complexity of mobility in Mexico City, the O-D matrix does not have to be symmetric nor the sum of the row *i* has to be equal to the sum of the column *i*. This can be explained because the O-D matrix captures chains of trips that may begin one day and end within the next few days. For instance, trips of people who leave their home to go to zone *i* to work and then go to zone *j* to see a movie before returning home, or people that work in zone *i* for few consecutive days (see [35]). In this paper, we consider all of the trips and they are identified by the mode of transport that was used in the area of interest (see Table 2).

**Table 2:** Transport modes used in the area of interest.

Car	RTP/M1	Metrobus/Mexibus	Motorcycle taxi
Collective/micro	Bicycle	Light rail	School transportation
Taxi app	Bus	Suburban	Personal transportation
Taxi	Motorcycle	Walk	Other
Subway	Trolleybus	Pedicab	

There are several methodologies to update the O-D matrix in the literature. Most of them combine known information with current data observed, such as the number of trips in some segments of the transit network [36]. There are also some approaches that project the trips to/from each zone based on the projected economic growth in those areas [37]. Nevertheless, given the pandemic situation that we are experiencing today and that we have current available data about the increase or decrease in mobility for some modes of transport, transit stations and parking lots, we consider the 2017 O-D matrix as a reference matrix and we update it to a scenario in 2020 using the daily mobility reports provided by Google [25] and the government of Mexico City [26]. According to [24], Tables 3 to 6 represent the number of trips between these zones; for instance, the mean number of trips whose origin is Coyoacán (id = 2) and destination is Iztapalapa (id = 6) during a week day is 228,272 (see Table 3) and the mean number of trips whose origin is Tláhuac (id = 10) and destination is Cuauhtémoc (id = 14) is 21,881 (see Table 6) during a day on the weekend.

**Table 3:** Mean number of trips from zones 1-16 to zones 1-9 during a week day.

Id	1	2	3	4	5	6	7	8	9
1	477560	21108	7564	120676	6475	25026	1557	0	24149
2	21111	886428	11010	48039	32230	228272	49305	17446	108741
3	7336	10462	301805	9770	5397	10867	3491	722	100180
4	120735	50796	9699	1677356	23427	52676	2602	590	27846
5	6486	32393	5358	22936	361938	181660	1563	145	19190
6	25244	221750	10308	56688	177845	2766471	9326	10395	64521
7	1685	50941	4148	2576	1389	8008	260789	441	83675
8	0	16022	722	590	145	10872	336	218175	3901
9	24180	108129	102892	28836	18012	61990	80171	5577	1034673
10	1730	50969	1994	7283	8243	134119	2179	22801	11070
11	11034	237367	10663	17958	14370	82757	53911	17203	95685
12	2148	100785	2637	6062	7586	46856	7030	31284	14993
13	17200	126058	14645	59944	59956	163732	29187	9714	161543
14	88252	118292	20292	264298	84725	253810	22415	9495	107543
15	99868	52246	43942	81013	26450	73629	11072	2972	115902
16	15795	31971	3324	103651	56192	102397	3239	1199	22583
Sum	920364	2115717	551003	2507676	884380	4203142	538173	348159	1996195

**Table 4:** Mean number of trips from zones 1-16 to zones 10-16 during a week day.

Id	10	11	12	13	14	15	16	Sum
1	1918	10139	2112	17283	93083	102450	16775	927875
2	48004	242818	103414	124379	118140	52754	34231	2126322
3	1391	11779	2118	16210	21397	42701	3179	548805
4	7446	18848	6419	60994	261785	81367	100561	2503147
5	7728	13951	7546	60375	85200	24730	58757	889956
6	133560	84369	42750	167620	260158	75002	100842	4206849
7	2086	50756	7950	29523	22641	11200	2466	540274
8	21893	18402	31875	10191	11670	3137	2059	349990
9	10788	99656	15231	164890	106156	120847	25640	2007668
10	517763	26394	50459	23977	26811	14303	7563	907658
11	22491	893910	124478	78302	89627	39742	15378	1804876
12	52346	121534	694220	33487	42853	13362	6662	1183845
13	22689	74181	32266	410876	150510	66226	41365	1440092
14	24894	89400	46227	145677	821467	151255	152044	2400086
15	13294	38943	12352	66648	149602	542212	37905	1368050
16	6620	14267	5001	39229	150324	36686	491938	1084416
Sum	894911	1809347	1184418	1449661	2411424	1377974	1097365	24289909

**Table 5:** Mean number of trips from zones 1-16 to zones 1-9 during a day of the weekend.

	1	2	3	4	5	6	7	8	9
1	331141	13688	3114	68471	5834	10607	1203	378	9658
2	11696	551750	4838	25849	19910	120115	30555	16952	52884
3	2174	4091	175522	3007	3271	7323	4482	492	65008
4	69138	24356	4142	1007667	16605	29428	825	874	11786
5	4545	20175	3729	16417	210228	128570	1118	793	8682
6	11046	124120	7325	32746	128502	1670601	4991	7800	43020
7	1311	29099	3224	1916	1200	4804	158791	3232	51829
8	252	13668	0	925	793	7594	1677	143275	2146
9	8721	52044	68219	13616	9358	40811	50897	3938	579592
10	1406	26844	757	5531	3253	100356	1831	13138	6297
11	6821	147822	2869	17403	8955	55210	32614	12158	55182
12	2622	58174	1034	4675	5281	27902	3341	25524	9751
13	10562	63766	7127	29544	39022	84688	15222	3119	85047
14	56596	66481	13774	184327	57030	180473	13321	8988	60055
15	53266	17612	17869	37333	13774	36801	4374	4100	59194
16	9800	19479	3206	72083	33526	67913	2627	3114	22571
Sum	581097	1233169	316749	1521510	556542	2573196	327869	247875	1122702

**Table 6:** Mean number of trips from zones 1-16 to zones 10-16 during a day of the weekend.

	10	11	12	13	14	15	16	Sum
1	1501	5786	1542	9701	63331	53846	11181	590982
2	26855	150388	60418	64149	67953	19583	19995	1243890
3	493	3714	856	8218	13863	18850	3176	314540
4	6370	16430	3851	28840	181498	37641	71621	1511072
5	3140	9292	8080	40741	56400	13737	33730	559377
6	99372	52020	30083	94294	181288	40162	68541	2595911
7	1013	37941	3235	14790	14315	4452	3892	335044
8	13264	10182	23451	2704	11629	3264	1256	236080
9	5619	54948	10135	87576	65512	60836	26414	1138236
10	345171	17204	50337	14740	21881	6563	5956	621265
11	15078	546694	77342	41336	58766	21318	10691	1110259
12	51898	76431	452836	17491	33051	8737	6028	784776
13	14753	38855	18884	232506	85671	28496	20327	777589
14	22350	60141	30310	84877	427114	92312	114306	1472455
15	7156	19593	10801	27493	94657	339935	28238	772196
16	6200	10326	5581	20049	116777	26215	344904	764371
Sum	620233	1109945	787742	789505	1493706	775947	770256	14828043

In Tables 3–6, the last row represents the total number of trips attracted by each zone and the last column in Tables 4 and 6 represents the total number of trips generated by each zone. This way we can see in Table 4 that during a week day 849,911 trips are attracted to zone 10 and 540,274 trips are generated from zone 7.

To compute the new number of trips made using the subway, RTP/M1, trolleybus, light rail or suburban, we used the corresponding rates given by the government of Mexico City. To compute the number of trips made using collective/micro or buses, we used the rates of transit stations given by Google and for personal transportation we used the workplaces rate. To compute the number of trips made by bicycle, we used the rates for Ecobici and for metrobus/mexibus we used an average of both the rates of metrobus and mexibus in Mexico City. The other transport modes remain the same as in 2017.

**Fig 2:**
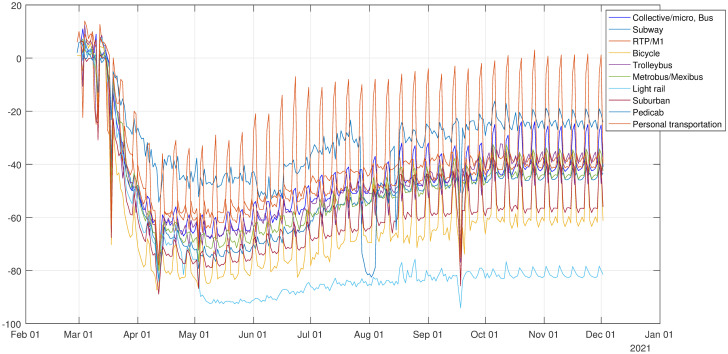
Variations in the mobility indices from 27 February, 2020 to 31 November, 2020.

Fig 2 shows the variations in the mobility indices from 27 February to 31 November, for each mode of transport that was modified.

The population of each borough is also considered in our mathematical model. According to [38], these populations in 2020 are given in Table 7.

**Table 7:** Population in 2020 for each borough of Mexico City.

Id	Borough	Population
1	Azcapotzalco	414,711
2	Coyoacán	628,063
3	Cuajumalpa de Morelos	199,224
4	Gustavo A. Madero	1,185,772
5	Iztacalco	384,326
6	Iztapalapa	1,815,786
7	La Magdalena Contreras	239,086
8	Milpa Alta	137,927
9	Álvaro Obreón	726,664
10	Tláhuac	305,076
11	Tlalpan	574,577
12	Xochimilco	407,885
13	Benito Juárez	385,439
14	Cuauhtémoc	531,831
15	Miguel Hidalgo	372,889
16	Venustiano Carranza	430,978

Note that both, the mobility rates from Google and the government of Mexico City and the reference O-D matrix (the one from 2017) are given per day. This way, the population *N*_*i*_ could be considered as constant; but only per day. In this work, a phenomenon whose duration is of the order of months is studied, so that for the entire period of estimation *N*_*i*_ is considered as a variable.

Furthermore, although in all boroughs the largest number of trips are carried out within the same borough, the distribution of trips to/from the other boroughs does not follow the same pattern in all cases. For example, the Coyoacán borough attracts the highest number of trips from the Tlalpan borough and the least amount of trips from Cuajimalpa; meanwhile, the borough of Iztapalapa attracts the highest number of trips from the Cuauhtémoc borough and the least amount of trips from La Magdalena Contreras. This phenomenon can be explained by the prevailing economic activity in each borough and the transportation connectivity with the others.

In order to obtain an stable algorithm for modeling correctly the migration of people, we use the Fratar method to balance all the origin-destination matrices [39].

### Clusters

In this section, we describe the clustering analysis that we implemented on Mexico City based on mobility data. This analysis is presented not only to try to find some possible socioeconomic relations between some boroughs, but because there exist computational challenges which can be avoided creating clusters. Firstly, in order to solve model (1) for the whole Mexico City, the program uses around 50GB in RAM during the compilation process using the Stan package. Secondly, the computation time for estimating the parameters is around 3 days. We have used a computer with Ubuntu 20.04, 64 GB in RAM and 12 cores. Therefore, we propose in this section how it could be avoided both of these computational challenges. Solving model (1) for each cluster would reduce the amount of RAM memory used and the computation time. Moreover, this strategy could be implemented simultaneously using the t-walk package for example, since the t-walk package only uses one core for execution program.

**Fig 3:**
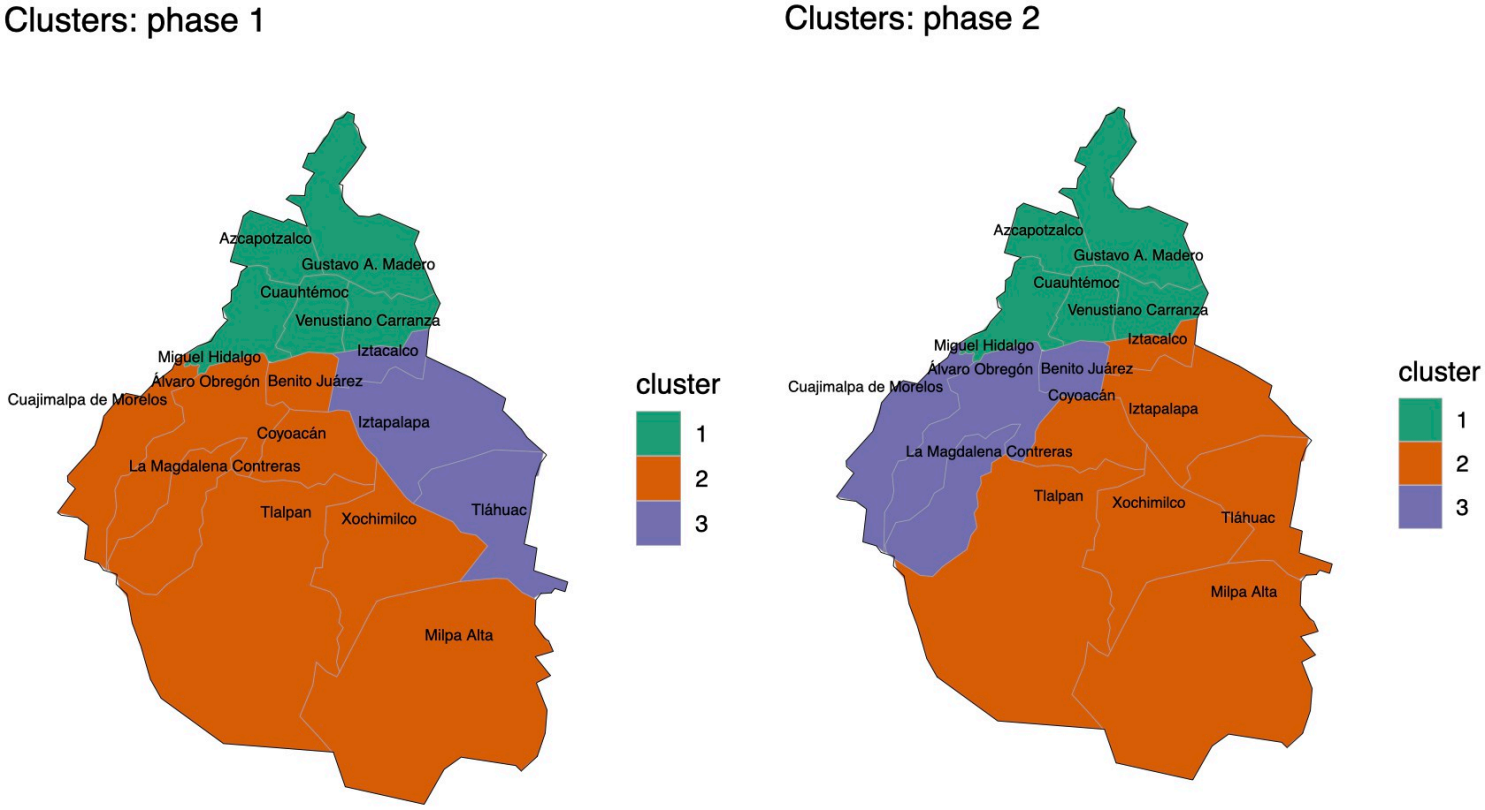
Borough clusters of Mexico City. (A): Borough clusters for the first period of the pandemic. (B): Borough clusters for the second period of the pandemic.

**Fig 4:**
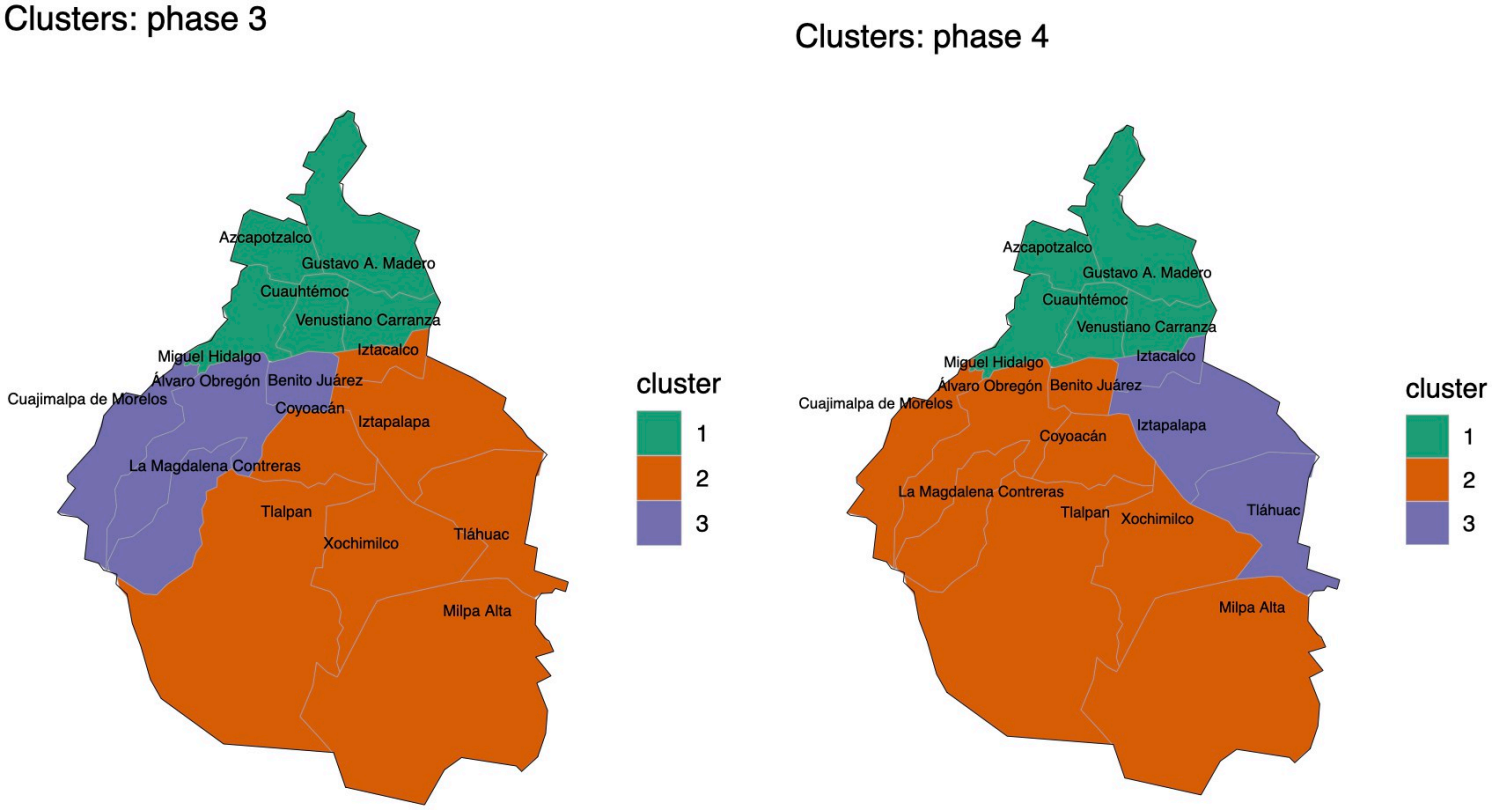
Borough clusters of Mexico City. (A): Borough clusters for the third period of the pandemic. (B): Borough clusters for the fourth period of the pandemic.

During the pandemic, the Mexican government has scheduled four phases depending of level of contagion risk. These phases corresponding to the following periods: phase 1: from 27 February February to 22 March; phase 2: from 23 March to 19 April; phase 3: from 20 April to 28 June; phase 4: from 29 June to 27 October. The mobility network was analysed using the community detection module Louvain inside the *igraph* R package [40]. For more details about the *igraph* R package, see [41]. Thus, using the Louvain community detection algorithm, we are able to identify that Mexico City’s network has a modular structure, with three communities, as shown in Figs 3 and 4. From Figs 3 and 4 we observe that the communities in the first and fourth phases of the pandemic are the same, and the second and third of the pandemic are the same. The community 1 of the first phase of the pandemic is composed of boroughs 1, 4, 14, 15 and 16; community 2 is composed of boroughs 2, 3, 7, 8, 9, 11, 12 and 13; and, community 3 is composed of boroughs 5, 6 and 10. The community 1 of the second phase of the pandemic is composed of boroughs 1, 4, 14, 15 and 16; community 2 is composed of boroughs 2, 5, 6, 8, 10, 11, and 12; and, community 3 is composed of boroughs 3, 7, 9 and 13.

## Mathematical model

As we mention in the introduction, the transmission model incorporates information on human movement within the following Susceptible, Exposed, Infected, Recovered (SEIR) metapopulation structure [21]:

$$\begin{array}{c} \left\{ \begin{array}{cc} & \frac{dS_{i}}{dt}=-\frac{\beta_{s}S_{i}I_{i}}{N_{i}}-\frac{\beta_{a}S_{i}A_{i}}{N_{i}}+\theta \sum_{j}\frac{M_{ij}S_{j}}{N_{j}-I_{j}}-\theta \sum_{j}\frac{M_{ji}S_{i}}{N_{i}-I_{i}}\\ & \frac{dE_{i}}{dt}=\frac{\beta_{s}S_{i}I_{i}}{N_{i}}+\frac{\beta_{a}S_{i}A_{i}}{N_{i}}-\alpha E_{i}+\theta \sum_{j}\frac{M_{ij}E_{j}}{N_{j}-I_{j}}-\theta \sum_{j}\frac{M_{ji}E_{i}}{N_{i}-I_{i}}\\ & \frac{dA_{i}}{dt}=\left(1-\rho \right)\alpha E_{i}-\gamma A_{i}+\theta \sum_{j}\frac{M_{ij}A_{j}}{N_{j}-I_{j}}-\theta \sum_{j}\frac{M_{ji}A_{i}}{N_{i}-I_{i}}\\ & \frac{dI_{i}}{dt}=\rho \alpha E_{i}-\gamma I_{i}\\ & N_{i}=N_{i}^{0}+\theta \sum_{j}M_{ij}-\theta \sum_{j}M_{ji},\;j=1,2,\ldots ,n, \end{array}\right. \end{array}$$ (1)

where *S*_*i*_, *E*_*i*_, *A*_*i*_, *I*_*i*_ and *N*_*i*_ are the susceptible, exposed, undocumented infected, documented infected and the total population in borough *i* at time *t*, respectively, and $N_{i}^{0}$ denotes the fixed population in borough *i* given by Table 7. Spatial coupling within the model is represented by the daily number of people traveling from city *j* to city *i* (*M*_*ij*_) an a multiplicative scale factor ***θ***, reflecting the under-reporting of human movement. It is also assumed that documented infected individuals (*I*_*i*_) do not move between boroughs, although these individuals can move between boroughs during the latency period. The total population *N*_*i*_ in each borough is reset each new day as the sum of $N_{i}^{0}$ and the inflow term *θ*∑_*j*_ *M*_*ij*_, minus the outflow term *θ*∑_*j*_ *M*_*ji*_. We note that distinction between daytime and nighttime in the transmission model 1 is implemented in [35]. A complete description of the parameters involved in the model (1), the respective range of values proposed in [21] and their measurement units can be found in Table 8. We have set a minimum value for the denominator *N*_*j*_ − *I*_*j*_ or *N*_*i*_ − *I*_*i*_ as equal to 10^3^ in order to avoid instabilities.

**Table 8:** Parameter description and values proposed in [21] of the state equations given on (1).

Parameter	Description	Proposed Range	Units
*β*_*s*_	Transmission rate due to documented infected individuals.	[0., 1.]	None
*β*_*a*_	Transmission rate due to undocumented individuals.	[0., 1.]	None
1/*θ*	Multiplicative factor (*reflect under-reporting of human movement*).	[0., 1.]	*Trips*/*person*
1/*α*	Average latency period.	[2, 10]	*Days*
1/*γ*	Average duration of infection.	[2, 20]	*Days*
*ρ*	Fraction undocumented/documented people.	[0.,.4]	None

The values are given in days as time unit.

## Parameter estimation

For parameter estimation, we use the daily reported dataset [23]. We use Bayesian inference to solve the inverse problem associated to the system of Ordinary Differential Equations (ODEs) given on (1), similarly to [33]. Some references using this method of parameter estimation can be found in [42–53].

Let us denote the vector of state variables in the zone *i* as **x** = (*S*_*i*_, *E*_*i*_, *A*_*i*_, *I*_*i*_) ∈ (*L*^2^[0, *T*])^*n*^, where *n* = 4 denotes the number of state variables and the vector of parameters in the zone *i* as $\theta =\left(\beta_{s},\beta_{a},\theta ,\rho ,\gamma ,\alpha ,S_{i_{0}},E_{i_{0}},A_{i_{0}},I_{i_{0}}\right)\in R^{m}$, where *m* = 10 denotes the dimension number of parameters to estimate. Thus, we can write the model (1) as the following Cauchy problem

$$\begin{array}{c} \left\{ \begin{array}{cc} & \dot{x}=\varphi \left(x,\theta \right)\\ & x\left(0\right)=x_{0}. \end{array}\right. \end{array}$$ (2)

Problem (2), defines a mapping Φ(***θ***) = **x** from parameters ***θ*** to state variables **x**, where $\Phi :R_{+}^{m}\to {\left(L^{2}(\left[0,T\right]\right)}^{n},$ where $R_{+}$ denotes the non-negative real numbers. We assume that Φ has a Fréchet derivative. Usually, not all states of the system can actually be directed measured, i.e., the data consists of measurements of some state variables at a discrete set of points *t*_1_, …, *t*_*k*_, e.g. in epidemiology, these data consist of number of cases of confirmed infected people. This defines a linear observation mapping from state variables to data $\Psi :{\left(L^{2}(\left[0,T\right]\right)}^{n}\to R^{s\times k}$, where *s* ≤ *n* is the number of observed variables and *k* is the number of sample points. Let us define $F:R^{m}\to R^{s\times k}$ as *F*(***θ***) = Ψ(Φ(***θ***)), called the forward problem. Thus, the inverse problem is formulated as a standard optimization problem

$$\begin{array}{c} \underset{\theta \in R^{m}}{\text{min}}{\left\|F\left(\theta \right)-y_{\text{obs}}\right\|}^{2}, \end{array}$$ (3)

such that **x** = Φ(***θ***) holds, with **y**_obs_ is the observable data which has error measurements of size ***η***.

Problem (3) may be solved using numerical tools to deal with a non-linear least-squares problem [54–58]. In this work, we implement Bayesian inference to solve the inverse problem given on (3). From the Bayesian perspective, all of the state variables **x** and parameters ***θ*** are considered as random variables and the data **y**_obs_ is fixed. For the random variables **x** and ***θ***, the joint probability distribution density of the data **x** and the parameters ***θ***, denoted by *π*(***θ***, **x**), is given by *π*(***θ***, **x**) = *π*(**x**|***θ***)*π*(***θ***), where *π*(**x**|***θ***)*π*(***θ***) is the conditional probability distribution, which is also called the likelihood function, and *π*(**x**|***θ***) is the prior distribution, which involves the prior information of parameters ***θ***. Given **x** = **y**_obs_, the conditional probability distribution *π*(***θ***|**y**_obs_), which is called the posterior distribution of ***θ***, is given by the Bayes’ theorem:

$$\begin{array}{c} \pi \left(\theta |y_{\text{obs}}\right)\propto \pi \left(y_{\text{obs}}|\theta \right)\pi \left(\theta \right), \end{array}$$ (4)

If an additive noise is assumed

$$\begin{array}{c} y_{\text{obs}}=F\left(\theta \right)+\eta , \end{array}$$

where ***η*** is the noise due to discretisation, the model error and the measurement error. If the noise probability distribution *π*_*H*_(***η***) is known, ***θ*** and ***η*** are independent, then

$$\begin{array}{c} \pi \left(y_{\text{obs}}|\theta \right)=\pi_{H}\left(y_{\text{obs}}-F\left(\theta \right)\right). \end{array}$$

All of the available information regarding the unknown parameter ***θ*** is codified into a prior distribution *π*(***θ***), which specifies our belief in a parameter before observing the data. All of the available information regarding how we obtained the measured data is codified into the likelihood distribution *π*(**y**_obs_|***θ***). This likelihood can be seen as an objective or cost function because it punishes deviations of the model from the data. To solve the associated inverse problem (4), one may use the maximum a posterior (MAP)

$$\begin{array}{c} \theta_{\text{MAP}}=\underset{\theta }{\text{max}\;}\pi \left(\theta |x\right). \end{array}$$

We used the dataset in the zone *i* as $y_{\text{obs}}=\left(\tilde{S_{i}},{\tilde{E}}_{i},{\tilde{A}}_{i},\tilde{I_{i}}\right)$, which correspond to the susceptible, exposed, documented infected and undocumented infected in the zone *i*, respectively. A Poisson distribution with respect to the time is typically used to account for the discrete nature of these counts. However, the variance of each component of the dataset **y**_obs_ is larger than its mean, which indicates that there is over-dispersion of the data. Thus, a more appropriate likelihood distribution is to use the Negative Binomial (NB) because it has an additional parameter that allows the variance to exceed the mean [50, 51, 59]. The NB is a mixture of Poisson and Gamma distributions, where the rate parameter of the Poisson distribution itself follows a Gamma distribution [59, 60]. We note that although there are different mathematical expressions for the NB depending on the author or source, they are equivalent. Because of this multiple representation of the NB in the literature, one must ensure to use the NB distribution accordingly to the source. Here, we have used the following expression for the NB distribution

$$\begin{array}{c} \text{NB}\left(y|\mu ,\phi \right)=\frac{\Gamma (y+\phi )}{\Gamma (y)\Gamma (\phi )}{\left(\frac{\mu }{\mu +\phi }\right)}^{y}{\left(\frac{\phi }{\mu +\phi }\right)}^{\phi }, \end{array}$$ (5)

where *μ* is the mean of the random variable y∼NB(y|μ,ϕ) and *ϕ* is the over-dispersion parameter; that is,

$$\begin{array}{cccc} E[Y] & =\mu , & \text{Var}(Y) & =\mu +\frac{\mu^{2}}{\phi }. \end{array}$$

We recall that the Poisson distribution has mean and variance equal to *μ*, so *μ*^2^/*ϕ* > 0 is the additional variance of the NB with respect to the Poisson distribution. The inverse of the parameter *ϕ*, controls the over-dispersion. Thus, it is important to select its support adequately for parameter estimation. In addition, there are alternative forms of the NB distribution. We have used the first option *neg_bin* of the NB distribution of Stan [61]. We acknowledge that some scientists have had success with the second alternative representation of the NB distribution [47]. We assume independent NB distributed noise ***η*** (i.e., all dependency in the data is codified into the contact tracing model). In other words, the positive definite noise covariance matrix ***η*** is assumed to be diagonal. Therefore, using the Bayes formula, the likelihood is

$$\begin{array}{c} \pi (\theta |\left(\tilde{I_{i}}\right)\propto \pi \left(\tilde{I_{i}}|\theta \right)\pi \left(\theta \right), \end{array}$$

where *i* denotes the borough index. As mentioned earlier, we approximate the likelihood probability distribution corresponding to diagnosed cases with a NB distribution

$$\begin{array}{c} {\tilde{I}}_{i}^{j}∼\text{NB}\left(I_{i}^{j}\left(\theta \right),\phi_{i}\right), \end{array}$$ (6)

where the index *j* denotes the number of days, *i* the number of the boroughs, and *ϕ*_*i*_ are the parameters corresponding to the over-dispersion parameter of the NB distribution (5) respect to each borough.

For independent observations, the likelihood distribution *π*(**y**|***θ***) is given by the product of the individual probability densities of the observations

$$\begin{array}{c} \pi \left(y_{\text{obs}}|\theta \right)=\prod_{j=1}^{n}\pi \left({\tilde{I}}_{i}^{j}|\theta \right), \end{array}$$

where the mean *μ* of the NB distribution $\text{NB}(I_{i}\left(\theta \right),\phi_{i})$, is given by the solution *I*_*i*_(*t*) of the model (1) at time *t* = *t*_*j*_. For the prior distribution, we select the LogNormal distribution for *β*_*s*_ and *β*_*a*_ parameters, Gamma distributions for *α* and *γ* parameters and Uniform distributions for the other parameters to estimate: *ρ*, ***θ*** and initial conditions $\left(S_{i_{0}},E_{i_{0}},A_{i_{0}},I_{i_{0}}\right)$. The hyperparameters and their support corresponding to all the distributions of the parameters to estimate are given on the table’s range Table 8.

$$\begin{array}{c} \begin{array}{cc} \pi (\theta ) & =\prod_{i=1}^{n}\text{LN}\left(a_{\beta },b_{\beta }\right)U\left(a_{q},b_{q}\right)U\left(a_{\delta },b_{\delta }\right)U\left(a_{\alpha },b_{\alpha }\right)U\left(a_{\gamma },b_{\gamma }\right)\\ & \;\times U\left(a_{s_{0}},b_{s_{0}}\right)U\left(a_{E_{0}},b_{E_{0}}\right)U\left(a_{I_{0}},b_{I_{0}}\right)U\left(a_{Q_{0}},b_{Q_{0}}\right). \end{array} \end{array}$$ (7)

The posterior distribution *π*(***θ***|*y*_obs_) given by (4) does not have an analytical closed form since the likelihood function, which depends on the solution of the non-linear model given on (1), does not have an explicit solution. Then, we explore the posterior distribution using two methods: first, the Stan Statistics package [61] within its version the Automatic Differentiation Variational Inference (ADVI) method,; and second, the general purpose Markov Chain Monte Carlo Metropolis-Hasting (MCMC-MH) algorithm t- walk [62]. Both algorithms generate samples from the posterior distribution *π*(***θ***|**y**_obs_) that can then be used to estimate marginal posterior densities, mean, credible intervals, percentiles, variances, and others. We the reader refer to [63] for a more complex description of the MCMC-MH algorithms.

**Fig 5:**
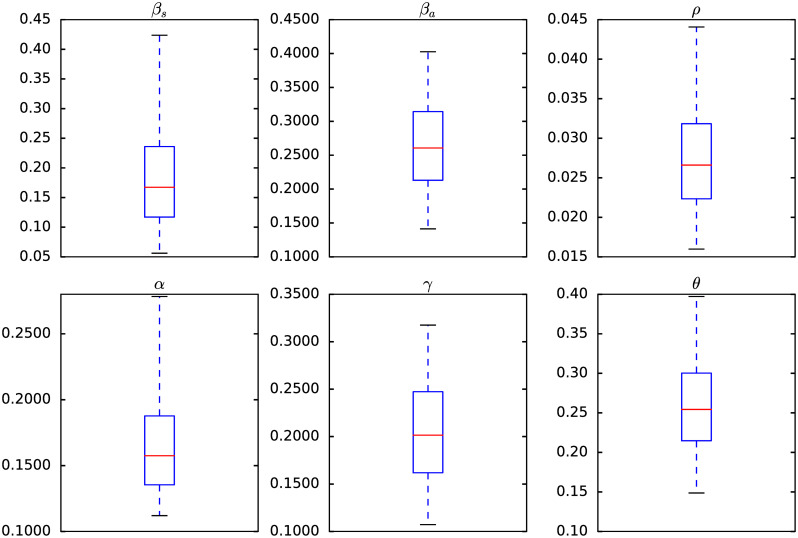
Credible intervals of parameters of the model (1) within 95% Highest-Posterior Density (HPD) using the Stan package [61].

**Fig 6:**
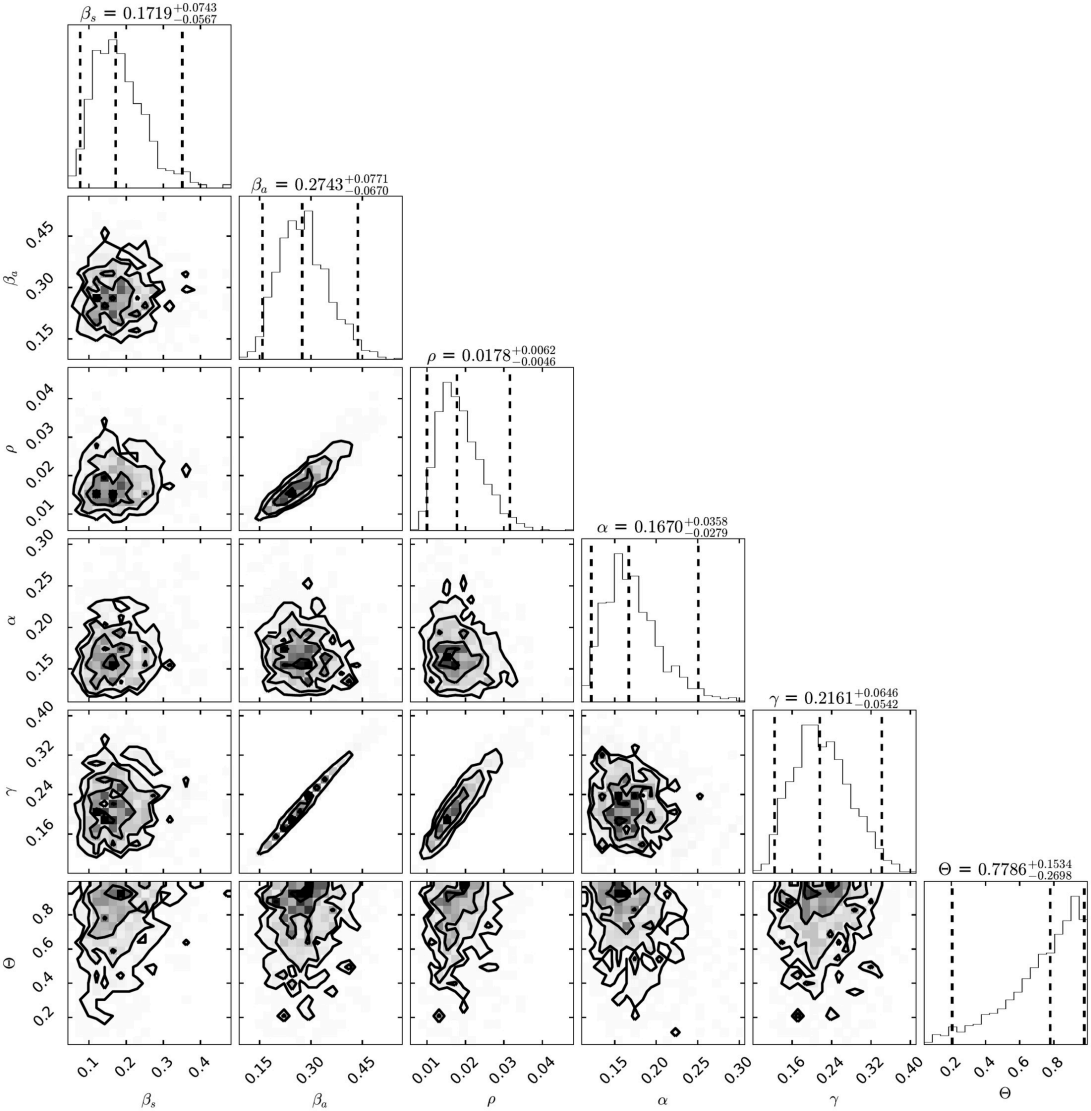
Joint probability density distributions of the estimated parameters of model (1) within 95% (HPD). The blue lines represent the medians.

**Fig 7:**
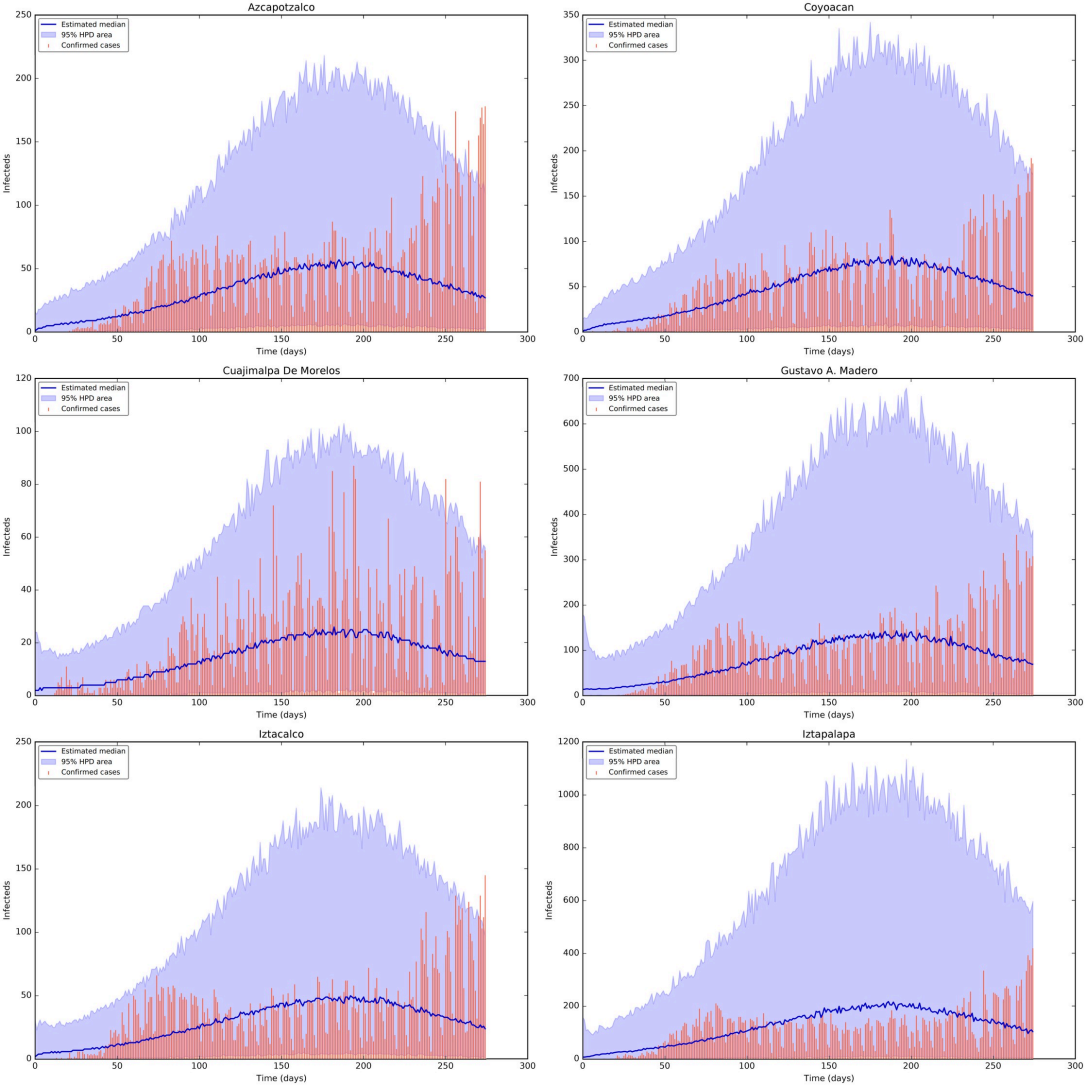
Fit of confirmed COVID-19 cases of the boroughs 1 to 6 using the Stan package [61]. Top row from left-hand to right-hand: the fit for the confirmed cases of the Districts Azcapotzalco and Coyoacan. The tomato colour bars represent the confirmed cases, the blue and purple solid lines represent the median and the mode, respectively, and the shaded area represent the %95 probability bands for the expected value for the state variable of Documented Infecteds. Middle row from left-hand to right-hand: the fit for the diagnosed cases of the Districts Cuajimalpa de Morelos and Gustavo A. Madero. Bottom row from left-hand to right-hand: the fit for the diagnosed cases of the Districts Iztacalco and Iztapalapa.

**Fig 8:**
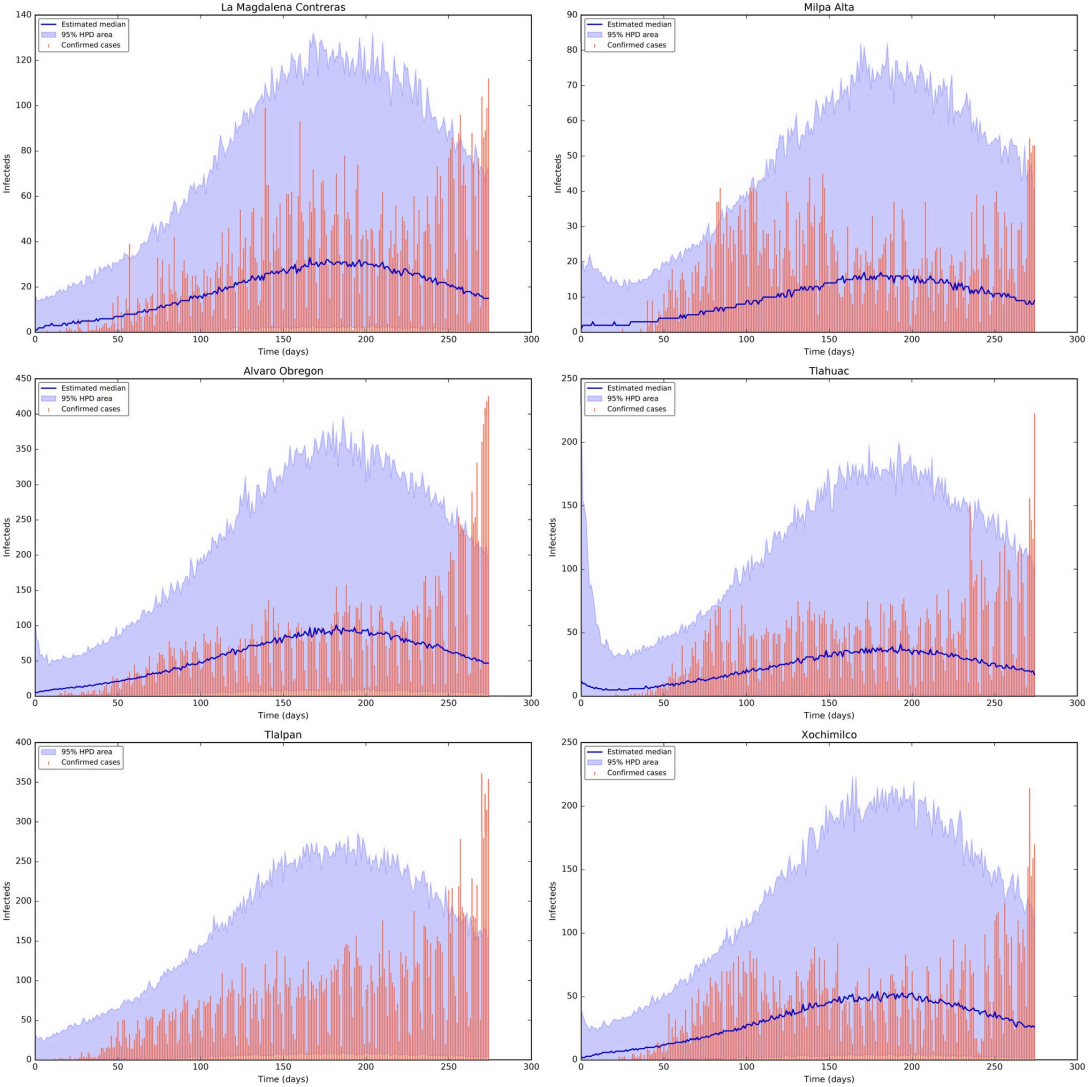
Fit of confirmed COVID-19 cases of the boroughs 7 to 12 using the Stan package [61]. Top row from left-hand to right-hand: the fit for the confirmed COVID-19 cases of the Districts La Magdalena Contreras and Milpa Alta. The tomato colour bars represent the confirmed COVID-19 cases, the blue solid line represent the median and the shaded area represent the %95 probability bands for the expected value for the state variable of Documented Infecteds. Middle row from left-hand to right-hand: the fit for the diagnosed cases of the Districts Alvaro Obregon and Tlahuac. Bottom row from left-hand to right-hand: the fit for the diagnosed cases of the Districts Tlalpan and Xochimilco.

**Fig 9:**
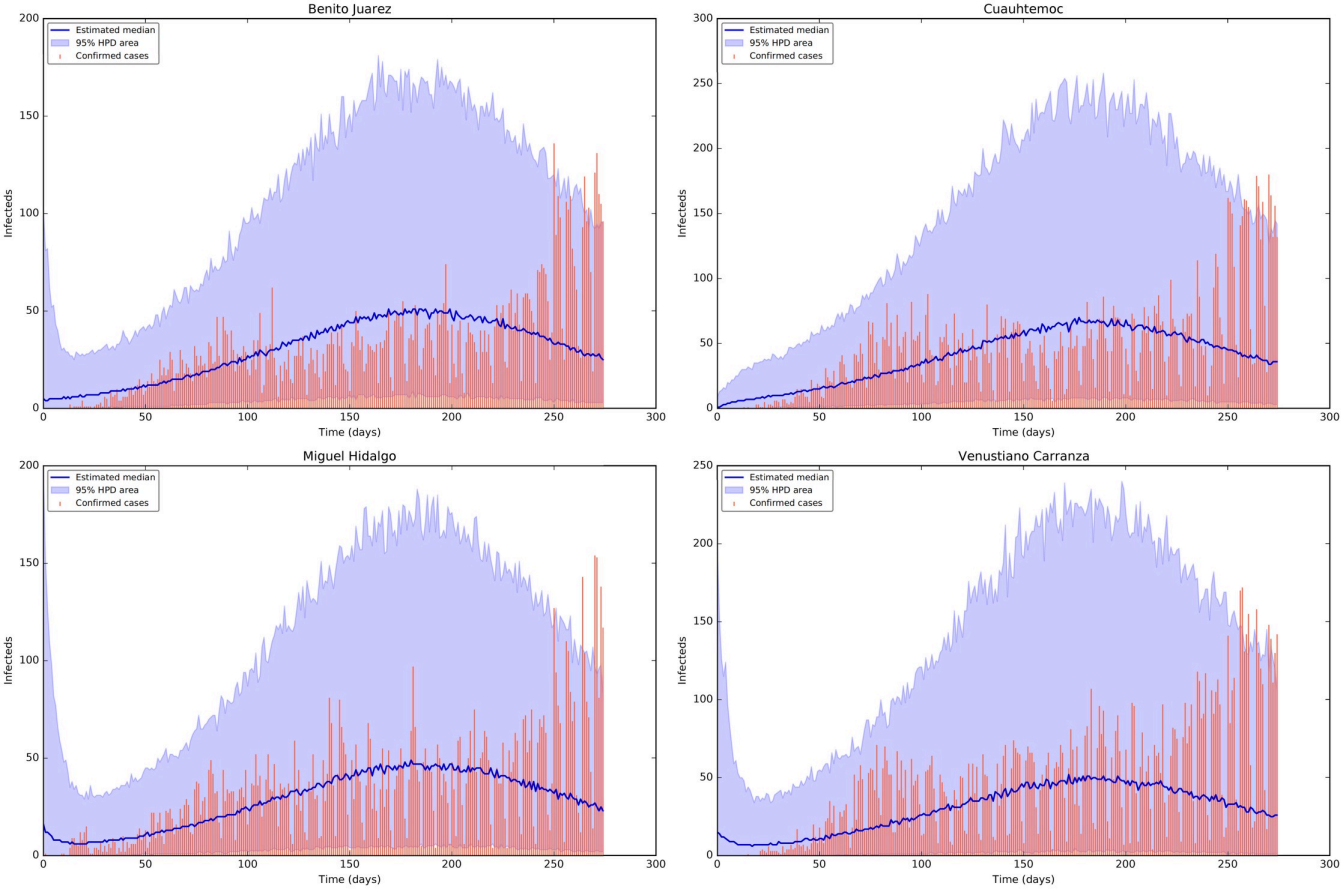
Fit of confirmed COVID-19 cases of the boroughs 13 to 16 using the Stan package [61]. Top row from left-hand to right-hand: the fit for the confirmed COVID-19 cases of the Districts Benito Juarez and Cuauhtemoc. The tomato colour bars reprent the confirmed COVID-19 cases, the blue solid line reprent the median and the shaded area represent the %95 probability bands for the expected value for the state variable of Documented Infecteds. Bottom row from left-hand to right-hand: the fit for the diagnosed cases of the Districts Miguel Hidalgo and Venustiano Carranza.

**Fig 10:**
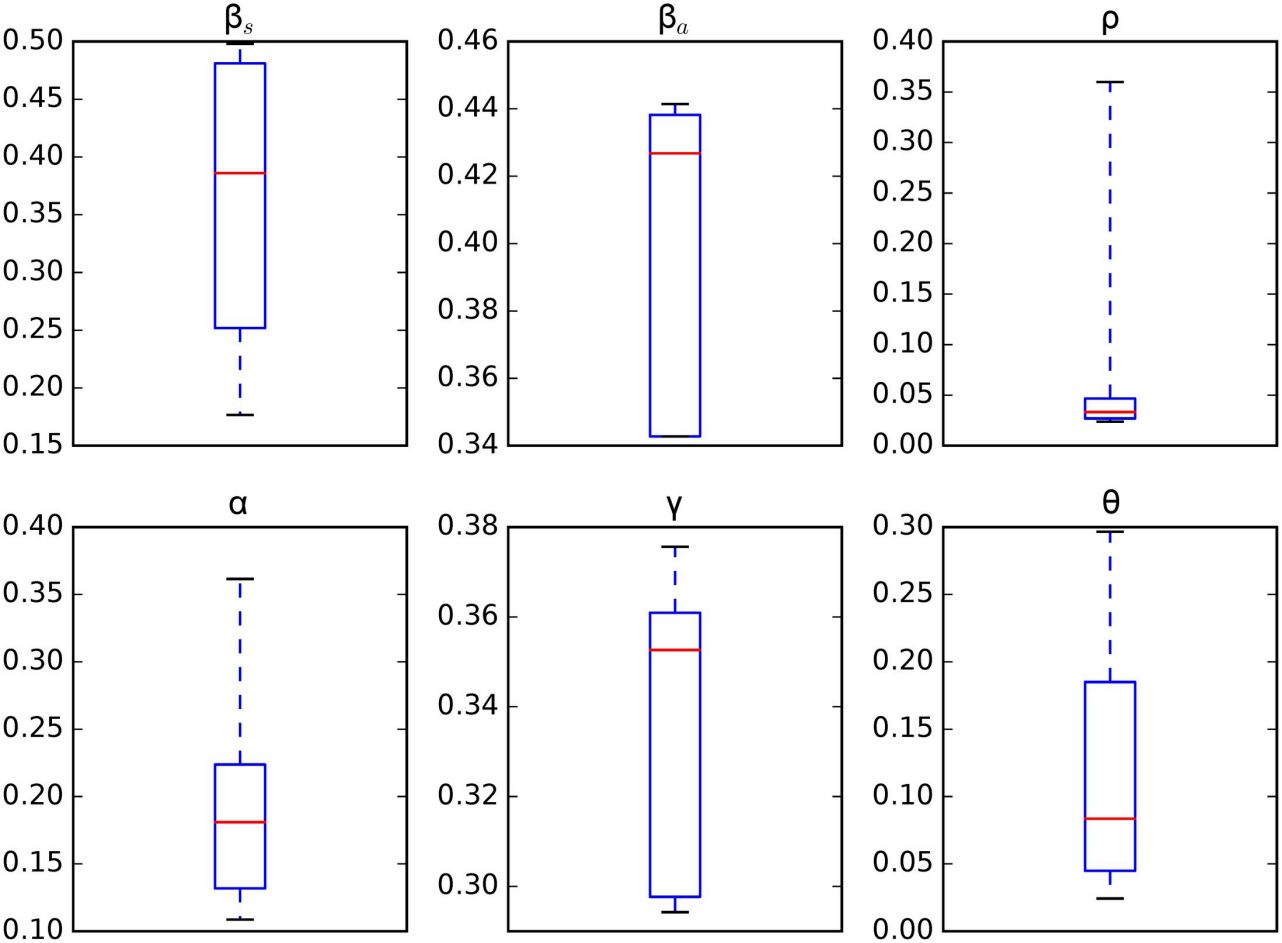
Credible intervals of parameters of model (1) within 95% Highest-Posterior Density (HPD) using the t-walk package [62].

Fig 5 shows the credible intervals of parameters of model (1) within 95% Highest-Posterior Density (HPD) using the ADVI-Fullrank method of Stan package [61]. Table 9 shows the posterior mean and quantiles of all the estimated parameters of model (1) using the ADVI-Fullrank method of Stan package. Table 10 shows the posterior mean and quantiles of all over-dispersion parameters *ϕ*_*i*_ of the Negative Binomial distribution (6). Fig 6 shows the joint probability density distributions of the estimated parameters of model (1) within 95% (HPD). The blue lines represent the medians. Figs 7–9 show the fit of confirmed COVID-19 cases of all of the boroughs of Mexico City using the Stan [61]. Fig 10 shows Credible intervals of parameters of model (1) within 95% Highest-Posterior Density (HPD) using the t-walk Package [62]. Note that the result obtained with the t-walk package are preliminary because we only performed 60,000 iterations, with 30,000 of them as burn-in and it was obtained without balancing the Origen-Destination matrices. We performed this limited quantity of iterations because the computational time consumption is significantly large for each 1,000 of iterations. However, we will perform more iterations in the near future. Using both packages, we did a fit for the first 245 days of the pandemic in Mexico City, starting 27 February, and we have performed predictions from 245–275 days, corresponding to 28 October to 27 November and compared with the true cases in this last period 28 October to 27 November. We assumed that the mobility from 28 October to 27 November is the same as from 28 September to 27 October, i.e., we assumed the same mobility cluster for the projection period. We set up a minimum borough fraction equal to 0.6 to limit the borough to fall below their population size. Our future work will analyse the identifiability of the parameters of model (1), as suggested in [49, 64, 65]. Specifically, the *ρ* parameter because it is multiplied by the period of incubation of the disease, *α*. Thus, estimating both parameters simultaneously may lead to non-identifiability difficulty. we have uploaded all the codes and source data used in this paper to the following Github link for a detailed review.

**Table 9:** Parameter estimation of *β*_*s*_, *β*_*a*_, *ρ*, *α*, *γ*, *θ* and initial conditions of the model (1).

	Mean	Std	Min	2.5%	50%	97.5%	Max
*β*_*s*_	0.1817	0.0688	0.0433	0.1299	0.1719	0.2215	0.4822
*β*_*a*_	0.2797	0.0724	0.0903	0.2274	0.2743	0.3259	0.5665
*ρ*	0.0186	0.0056	0.0057	0.0146	0.0178	0.0220	0.0481
*α*	0.1720	0.0334	0.1103	0.1485	0.1670	0.1910	0.3067
*γ*	0.2210	0.0570	0.0804	0.1798	0.2161	0.2597	0.4116
*θ*	0.7279	0.2112	0.0409	0.6113	0.7786	0.8976	0.9963
*E*_10_	215.5595	165.6988	9.4025	89.7281	172.8620	297.3270	901.8550
*A*_10_	546.1551	275.5846	13.5082	310.5730	565.1880	793.2150	995.1790
*I*_10_	1.9900	2.0874	0.0481	0.6862	1.3693	2.6035	22.6618
*E*_20_	253.3650	193.9120	3.8161	102.6910	201.1370	366.7250	936.9310
*A*_20_	295.6783	195.3005	9.7637	135.7270	260.0070	417.1160	873.4510
*I*_20_	3.4961	4.5445	0.0385	0.9100	1.9425	4.0675	40.6196
*E*_30_	185.7097	145.4584	1.9960	78.6950	147.7400	247.2920	835.4920
*A*_30_	507.7907	263.5192	6.1474	283.0790	514.8280	736.8860	984.3130
*I*_30_	4.2297	5.8422	0.0378	0.9611	2.2060	4.9589	61.0710
*E*_40_	683.1201	253.4763	18.5321	517.4730	743.1590	900.7840	999.2030
*A*_40_	496.4247	271.0262	6.0559	258.5220	495.7330	728.1480	987.1500
*I*_40_	30.8663	29.6572	0.0269	5.2495	19.7807	52.1318	99.6383
*E*_50_	552.5027	299.4180	4.4051	284.7780	577.9160	837.3670	999.1490
*A*_50_	562.7039	282.2821	1.3356	315.3790	605.4310	809.2500	997.2760
*I*_50_	4.9965	6.9620	0.0388	1.0097	2.4918	6.0742	61.8987
*E*_60_	450.4365	279.1041	1.4010	200.7940	420.6680	688.3850	992.9970
*A*_60_	547.0965	270.4777	9.5825	315.7010	568.5270	783.6100	992.2320
*I*_60_	23.4015	26.0301	0.0173	3.7063	12.1013	36.0308	99.6128
*E*_70_	115.0333	110.9839	2.8503	38.3297	76.6577	151.7080	795.5370
*A*_70_	515.9971	278.4386	6.2676	281.7120	534.1290	755.6920	997.6560
*I*_70_	3.7288	6.0063	0.0180	0.6917	1.6631	4.0766	55.2651
*E*_80_	224.1849	186.9898	3.0370	75.5207	172.3130	321.0610	925.9820
*A*_80_	569.0899	287.3211	1.8729	328.3040	597.3180	833.6790	999.2210
*I*_80_	3.4849	4.8696	0.0357	0.7935	1.7130	4.2425	44.0485
*E*_90_	188.5786	164.5613	3.3092	61.5219	134.4440	269.3350	807.6380
*A*_90_	687.0370	275.9913	10.8989	500.1660	765.8410	926.9680	999.2580
*I*_90_	16.5153	20.7336	0.0258	2.3711	7.4698	22.8262	96.2754
*E*_100_	356.7580	263.3986	2.5233	125.7860	297.0560	550.1360	987.3250
*A*_100_	746.1666	233.5140	56.2971	610.6280	829.0390	934.2400	999.0530
*I*_100_	31.6034	32.8951	0.0017	3.3353	16.6582	55.7096	99.9472
*E*_110_	281.1917	236.3608	2.7780	90.8408	206.4170	432.5200	970.9040
*A*_110_	252.0265	216.5767	1.4617	74.8393	192.5350	375.3250	980.7710
*I*_110_	4.0954	5.9613	0.0178	0.8408	1.9202	4.5660	54.8201
*E*_120_	130.6564	131.6714	2.0295	36.7382	85.9181	175.2460	823.6720
*A*_120_	189.1367	168.3434	3.8207	62.8391	135.1090	254.0920	847.2030
*I*_120_	8.7603	12.7619	0.0338	1.1913	3.9130	10.2037	94.1881
*E*_130_	135.1793	130.8547	2.4587	39.7409	89.4389	187.5800	786.0350
*A*_130_	233.7060	218.5237	2.5230	63.5877	160.4860	337.5610	966.4870
*I*_130_	17.2452	23.4479	0.0013	1.2447	6.5493	23.5534	99.6969
*E*_140_	288.4052	241.8465	2.0949	85.1094	216.5890	441.7050	967.2260
*A*_140_	132.8599	136.0876	1.5013	36.5287	86.8088	176.9500	817.9200
*I*_140_	1.1226	1.1440	0.0290	0.4021	0.7689	1.4526	13.1217
*E*_150_	194.1867	170.3316	2.6126	63.5594	138.1430	273.0570	818.0280
*A*_150_	629.8427	301.4854	5.1016	382.9950	700.1620	909.0470	999.4200
*I*_150_	31.9147	32.6907	0.0114	3.2822	18.5268	56.7282	99.9608
*E*_160_	615.1693	319.6856	0.5475	323.0720	703.2440	916.7530	999.8430
*A*_160_	590.1725	297.5308	7.9860	345.1590	634.7790	868.4580	999.4370
*I*_160_	34.3333	34.6819	0.0100	3.8419	18.8559	64.8812	99.9809

**Table 10:** Over-dispersion parameters estimation of the Negative Binomial distribution (6).

	Mean	Std	Min	2.5%	50%	97.5%	Max
*ϕ*_1_	2.1991	0.3290	1.2822	1.9601	2.1692	2.4130	3.5188
*ϕ*_2_	1.8943	0.3639	1.0883	1.6340	1.8487	2.1194	3.9229
*ϕ*_3_	2.0723	0.3572	1.2465	1.8068	2.0520	2.2942	3.2018
*ϕ*_4_	1.4408	0.2664	0.8347	1.2476	1.4203	1.6004	3.0505
*ϕ*_5_	1.8577	0.2999	1.1289	1.6330	1.8409	2.0425	2.9154
*ϕ*_6_	1.2810	0.2120	0.6839	1.1406	1.2595	1.4009	2.1667
*ϕ*_7_	2.0110	0.3294	1.0595	1.7742	1.9821	2.2169	3.3500
*ϕ*_8_	1.5453	0.3538	0.7403	1.2915	1.5145	1.7583	3.3937
*ϕ*_9_	2.1633	0.4467	1.1252	1.8410	2.1092	2.4173	4.3067
*ϕ*_10_	1.3869	0.2924	0.6578	1.1816	1.3610	1.5593	2.4075
*ϕ*_11_	2.4184	0.4398	1.1707	2.1076	2.3709	2.6832	4.0595
*ϕ*_12_	1.8298	0.4035	0.8828	1.5461	1.7847	2.0668	4.1388
*ϕ*_13_	2.6204	0.6058	1.2449	2.2169	2.5623	2.9725	6.2347
*ϕ*_14_	2.1933	0.3804	1.3056	1.9111	2.1554	2.4154	3.8624
*ϕ*_15_	2.1977	0.4987	1.0955	1.8362	2.1534	2.5010	3.9972
*ϕ*_16_	1.6167	0.3232	0.8505	1.3926	1.5958	1.8085	3.0021

## The basic reproduction number estimation

The basic reproduction number, which is commonly denoted by $R_{0}$, is the average number of secondary infections generated by a single infective during the curse of the infection in a whole susceptible population. We calculate the reproductive number $R_{0}$ in Mexico City using the inferred parameters. Define **X** = (*E*, *A*, *I*) and using the next generation operator method [66] on the system (1), the Jacobian matrices F and V of system (1) are given by

$$\begin{array}{c} F=(\begin{array}{c} \frac{\beta_{s}SA}{N}+\frac{\beta_{a}SI}{N}\\ 0\\ 0 \end{array})\;\text{and}\;V=(\begin{array}{c} \alpha E\\ \gamma A-\rho \alpha E\\ \gamma I-(1-\rho )\alpha E \end{array}). \end{array}$$

The disease free equilibrium (DFE) of system (1) is **X**_0_ = (0, 0, 0, *N*, 0)^*T*^, we then have

$$\begin{array}{c} F=\frac{\partial F}{dX}\left(X_{0}\right)=(\begin{array}{ccc} 0 & \beta_{s} & \beta_{a}\\ 0 & 0 & 0\\ 0 & 0 & 0 \end{array}) \end{array}$$

and

$$\begin{array}{c} V=\frac{\partial V}{dX}\left(X_{0}\right)(\begin{array}{ccc} \alpha & 0 & 0\\ -\rho \alpha & \gamma & 0\\ -(1-\rho )\alpha & 0 & \gamma \end{array}). \end{array}$$

Therefore, the next-generation matrix is **K** = **FV**^−1^, from where *R*_*e*_ is computed as the leading eigenvalue of matrix **K**; that is,

$$\begin{array}{c} R_{0}=\frac{\rho \beta_{s}}{\gamma }+\frac{\left(1-\rho \right)\beta_{a}}{\gamma }. \end{array}$$ (8)

Table 8 shows the range of values for the parameters involved in the expression (8) obtained using the Stan package [61]. With those values, we obtain a %95 credible interval for $R_{0}\in \left(1.0224,1.2376\right)$.

## Discussion

In this work, we analyse a networked dynamic metapopulation model of the coronavirus dissemination in Mexico City using ODEs and Bayesian statistics. We present an explanation of how to estimate the mobility per day between the boroughs that compound the Mexico City, both on a weekday and on weekends; combining available information from the origin-destination survey carried out in 2017 with the current mobility indices that Google and the government of Mexico City report, depending on the mode of transport used to make each trip (e.g., bus, subway, car, etc.) and then we use the Fratar method to balance the daily origin-destination matrices. We also present a clustering analysis of the boroughs which compound Mexico City based on mobility data from Google and the Transportation Mexican Survey. From Figs 3 and 4, we can identify three different clusters during the each phase of the pandemic. We point out that the same cluster analysis done for Mexico City, could be implemented for a broader area, the metropolitan area named Valle de Mexico, which is rather important for the whole country. We consider that this clustering analysis which is based on individual movement may be crucial to efficiently model a human pandemic on the same scale as presented here, or at a smaller scale.

From Fig 5, the transmission rate of symptomatic was 0.19 within 95% Credible Interval (CI) [0.06, 0.42], and the transmission rate of asymptomatic was 0.27 within 95% CI [0.14, 0.40], which is in concordance with the value estimated of 0.25 in [67], in that study the transmission rate was not separated in symptomatics and asyntomatics. The fraction of undocumented infections, *ρ*, was 0.027 within 95% CI [0.02, 0.04]. The estimated latency period, 1/*α*, is 5.96 days within 95% CI [3.60, 8.93] days, which is in concordance with the value used of 5.99, 6.0, 5.1 and 5.0 in [50, 52, 68, 69], and the estimated recovery period, 1/*γ*, is 4.86 days within 95% CI [3.15, 9.33] days, which is lower in comparison with the ones used of 5.97 and 10.81 (asymptomatic and symptomatic class, respectively) in [69], 10.0 in [52], 7.0 in [50] and 5.0, 10.8 and 14.0 (reported infectious, symptomatic and asymptomatic class, respectively) in [68]. The inter-borough scale factor ***θ*** was 0.77 within 95% CI [0.61, 0.89], this value indicates that the mean number of trips made by a person is between three and four in one day, which makes sense with complete trips to get out of home, do some activities (e.g., work, shopping, or services), and return home. The results of the inferred parameters of model (1) and the population size of the boroughs (e.g., Iztapalapa and Gustavo A. Madero) help to explain the fast dispersion of COVID-19 and indicate the challenge of finding strategies to contain it. We have compared the parameter values inferred with respect to those used for Mexico City. As mentioned in Section, we will analyse the identifiability of the parameters of model (1) (i.e., the *ρ* parameter) because this parameter is multiplied by the period of incubation of the disease, *α*. Thus, estimating both parameters simultaneously may lead to a non-identifiability difficulty. We may observe this non-identifiability in Figs 5 and 10; that is, different combinations of the model parameters lead to the same “energy” value of the system 1. In particular, we can observe that a different combination of the estimated parameters values obtained with the methods ADVI-Fullrank and ADVI-Meanfield give very similar fitted curves for diagnosed cases. We also observe that the recovery period time is more in accordance with the values used in [50, 52, 68, 69] but the latency period is lower than the ones used there. We note that the parameters, *β*_*s*_, *β*_*a*_ of the model (1) are considered as global; that is, they are assumed the same for all the boroughs of Mexico City and all the transportation modes. In the near future, we will explore a more robust model that will consist of local parameters of transmission *β*_*s*_, *β*_*a*_, instead of globally (i.e., a pair of transmission rates *β*_*s*_, *β*_*a*_ for each borough). Furthermore, we will consider those transmission rates, *β*_*s*_, *β*_*a*_, to be dependent on time as in [52, 70]. In addition, we will consider the interstate and international mobility from/to Mexico City. We will take into account imported cases from the other 31 states of Mexico. We will also consider the cases imported from overseas by airplane passengers, and will do a global and local sensitivity analysis of model (1). Finally, we will investigate a spatio-temporal model based on a diffusion partial differential equation model combined with individual movement trends.
